# Multilevel congenital ureteral obstruction in a two-year-old child: Staged reconstruction with neo-implantation and V-Y pyeloplasty

**DOI:** 10.1016/j.eucr.2026.103358

**Published:** 2026-01-27

**Authors:** Gede Wirya Kusuma Duarsa, Made Bagus Ari Pandita, Desak Agung Istri Padma Putri, Ronald Sugianto

**Affiliations:** a Department of Urology, Faculty of Medicine, Universitas Udayana, Prof IGNG Ngoerah General Hospital, Denpasar, Indonesia; b Department of Surgery, Faculty of Medicine, Universitas Udayana, Prof IGNG Ngoerah General Hospital, Denpasar, Indonesia; c Medical Doctor Study Program, Faculty of Medicine, Universitas Udayana, Prof IGNG Ngoerah General Hospital, Denpasar, Indonesia; d Department of Urology, Faculty of Medicine, Universitas Airlangga, Dr. Soetomo General Academic Hospital, Surabaya, Indonesia

**Keywords:** Congenital ureteral anomalies, Pediatric hydronephrosis, Ureteral reimplantation, Ureterovesical junction obstruction, Ureteropelvic junction obstruction

## Abstract

Multilevel congenital ureteral obstruction involving the ureteropelvic junction (UPJ) and ureterovesical junction (UVJ) is exceedingly rare condition in pediatric urology and may cause progressive hydronephrosis and renal impairment if untreated. We report a two-year-old boy with antenatally detected right-sided hydronephrosis caused by obstruction involving the UPJ, mid-ureter, and UVJ, previously managed with nephrostomy. Magnetic resonance urography demonstrated massive hydronephrosis and delayed renal excretion. The reconstructive surgery using non-dismembered V–Y pyeloplasty and ureteral neo-implantation with double-J stenting successfully restored urinary continuity and preserved renal function. This staged, anatomy-based strategy appears safe and effective for multilevel congenital ureteral obstruction.

## Highlights

• Rare multilevel ureteral obstruction involving UPJ, mid-ureter, and UVJ.
• Severe hydronephrosis detected antenatally with progressive enlargement.
• MRI showed high-grade obstruction with organ displacement and poor drainage.
• Staged V-Y pyeloplasty and ureteral neo-implantation restored urine flow.
• Personalized reconstruction effective for complex congenital ureteral disease.

## 1 Introduction

Congenital ureteral anomalies, particularly superimposed obstructions involving both the ureteropelvic junction (UPJ) and the ureterovesical junction (UVJ), constitute a rare and complex group of pediatric urologic anomalies. These anomalies can lead to progressive hydronephrosis, impaired renal function, and recurrent urinary tract infections if not managed appropriately.^1^ Antenatal ultrasound screening has significantly improved the early detection of these anomalies; however, the severity, location, and extent of the obstruction often require a comprehensive postnatal evaluation and staged surgery.^2^

Management of these cases is particularly challenging in young children due to the small anatomic size, increased risk of postoperative complications, and the need for an individually tailored reconstructive strategy. Simultaneous pyeloplasty and ureteral reimplantation are rarely reported in pediatric patients with multisegmented ureteral stenosis.^3^

We report a rare case of a two-year-old boy with multilevel congenital ureteral obstruction involving the ureteropelvic junction, mid-ureter, and ureterovesical junction, who presented with severe hydronephrosis detected antenatally and confirmed by postnatal imaging. The patient underwent staged reconstructive surgery, culminating in ureteral neo-implantation and a non-dismembered V–Y pyeloplasty with Double J (DJ) stent placement, and made a good postoperative recovery in the Pediatric High Care Unit.

## 2 Case presentation

A two-year-old boy was admitted to the Department of Urology for definitive management of congenital urinary tract obstruction. The urinary tract abnormality was first detected antenatally through a routine prenatal ultrasound, which revealed right-sided hydronephrosis that continued to enlarge throughout the pregnancy. Since birth, the parents had observed increasingly pronounced right-sided abdominal enlargement. Despite this, the patient maintained a normal voiding pattern with clear yellow urine and no history of dysuria, hematuria, urinary retention, or urinary tract infections.

The patient had undergone a right percutaneous nephrostomy in June 2024, then a month later, the nephrostomy was replaced due to catheter dysfunction and persistent hydronephrosis. The procedure was performed to relieve urinary tract obstruction and preserve kidney function. Antenatal and perinatal history revealed that the patient was the second child of a 34-year-old mother, born at 37 weeks of gestation by cesarean section due to obstetric complications, including secondary primigravida, oblique fetal position, polyhydramnios suspected to be due to a congenital anomaly (duodenal atresia), and mild microcytic hypochromic anemia. The baby weighed 3100 g, was 51 cm long, and had Apgar scores of 7 and 8 at the first and fifth minutes, respectively. The baby cried spontaneously immediately after birth and did not require further neonatal resuscitation.

Upon admission, the patient was asymptomatic and was scheduled for elective surgery as part of a staged reconstruction plan following a previous urinary diversion procedure. The child showed no signs of shortness of breath or gastrointestinal disturbances; his bowel movements remained regular.

Preoperative physical examination revealed the patient to be in good general condition, fully conscious, and hemodynamically stable. Body weight was 15 kg, height 75 cm. There were no signs of anemia or jaundice. A right flank examination revealed a nephrostomy tube with a sterile dressing. There was no leakage or erythema around the insertion site; urine appeared to drain well into the diaper. The suprapubic area was flat and non-tender. The external genitalia appeared male with good spontaneous voiding.

**Fig. 1:**
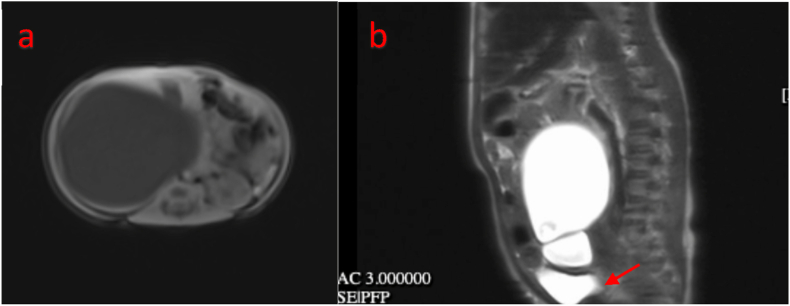
Preoperative contrast-enhanced magnetic resonance urography shows giant right hydronephrosis with severe dilation of the renal pelvis and proximal ureter extending into the pelvic region, and demonstrates delayed contrast enhancement in the right kidney (a) Axial view of non-contrast MR-urography (b) Sagittal view of Delayed Phase MR-urography.

**Fig. 2:**
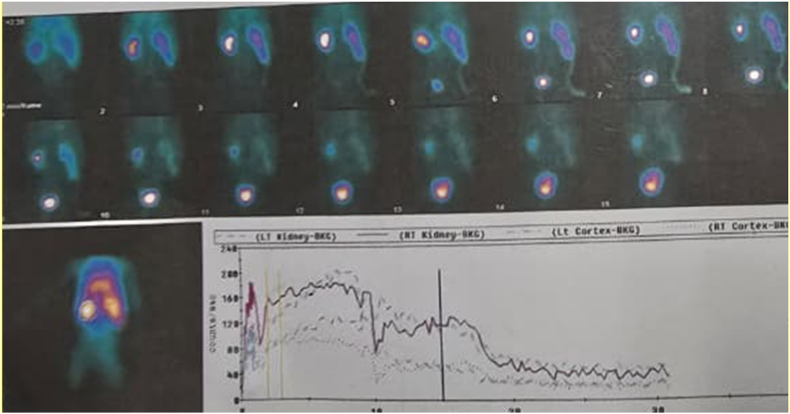
Renography showed good renal function for both kidneys. The right kidney function was 54.56 %, while the left kidney was 45.44 %.

A urinary ultrasound (August 19, 2025) showed severe hydronephrosis and hydroureter in the right kidney, while the left kidney and bladder appeared normal. A DJ stent was visible with the proximal end in the right renal pelvis, but the distal end was not visible in the bladder, indicating obstruction at the ureterovesical junction (UVJ). A chest radiograph (June 7, 2024) showed normal cardiac and pulmonary fields, but a soft tissue shadow was visible in the right upper quadrant of the abdomen pushing the intestine to the left, suggesting a mass effect due to enlargement of the right renal collecting system. Magnetic resonance urography (August 25, 2023) with contrast revealed a congenital obstructive megaureter at the right ureterovesical junction (UVJ) at the level of the S5 vertebra, accompanied by severe hydronephrosis displacing the liver superiorly and the intestine posterolaterally to the left, as shown in Fig. 1. The right kidney showed impaired excretory function, while the left kidney appeared normal. Renography (November 6, 2024) showed good right kidney function with hydronephrosis and no signs of obstruction. Left kidney function was good and there were no signs of obstruction, as shown in Fig. 2.

**Fig. 3:**
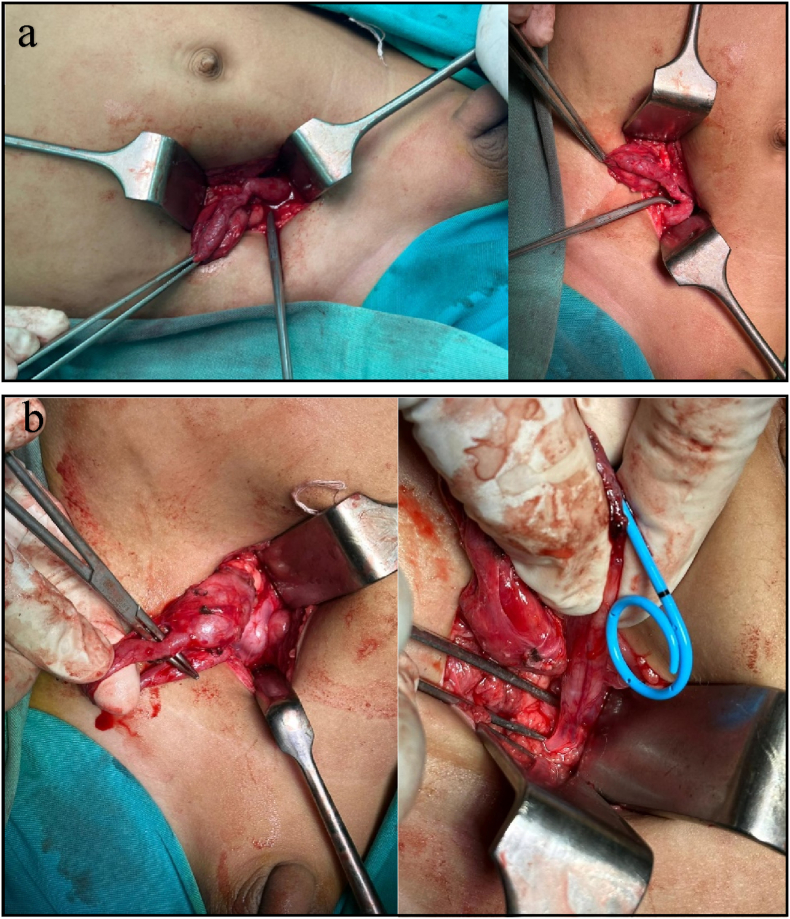
Durante operation **(a)** dilation of the right ureter and renal pelvis with multiple areas of narrowing along its course—including at the ureteropelvic junction (UPJ), mid-ureter, and ureterovesical junction (UVJ). **(b)** Definitive reconstruction was performed with a non-dismembered V–Y pyeloplasty at the ureteropelvic junction.

On October 9, 2025, the patient underwent surgery under general anesthesia and orotracheal intubation in the supine position. After routine skin preparation and sterile draping, the procedure began with a cystoscopy, which revealed a completely occluded right ureteral orifice, preventing stent placement. Open exploration through a right Gibson incision was then performed. Dissection revealed a significantly dilated right ureter proximally, with several narrowing segments (strictures) along its course—particularly at the mid-ureter, ureteropelvic junction (UPJ), and ureterovesical junction (UVJ), as shown in Fig. 3a. The renal pelvis and proximal ureter appeared enlarged and thickened, suggesting chronic obstruction. A biopsy of a segment of the ureter was performed for histopathology, to assess for congenital smooth muscle abnormalities or fibrotic changes.

The reconstruction procedure was performed with a V-Y non-dismembered pyeloplasty at the ureteropelvic junction, aimed at widening the narrowed area and restoring continuity between the renal pelvis and ureter. Next, ureteral neo-implantation with the Politano-Leadbetter method was performed into the bladder to re-establish a functional ureterovesical connection. The mid-ureter stricture was excised, and a tailoring from the proximal ureter to the distal ureter was performed. A DJ stent was placed for internal drainage, and a closed suction drain was placed in the right inguinal region, as shown in Fig. 3b. After hemostasis was achieved, the surgical wound was closed in layers according to anatomy. The procedure was uneventful without significant bleeding.

Post-operatively, the patient was transferred to the Pediatric High Care Unit (PHCU) for intensive monitoring. Upon arrival, the patient was conscious, calm, and hemodynamically stable with a blood pressure of 103/60 mmHg, a pulse rate of 118 beats per minute, a respiratory rate of 30 breaths per minute, and a body temperature of 36 °C. Urine output reached 250 mL within 4 hours postoperatively (equivalent to 4.1 mL/kg/hour) through a 10 Fr Foley catheter, indicating good renal perfusion and drainage. The nephrostomy site was maintained in a sterile condition with no leaks or signs of infection. The surgical wound appeared clean with a sterile dressing without bleeding or discharge, and the drain was draining a small amount of serohemorrhagic fluid.

**Fig. 4:**
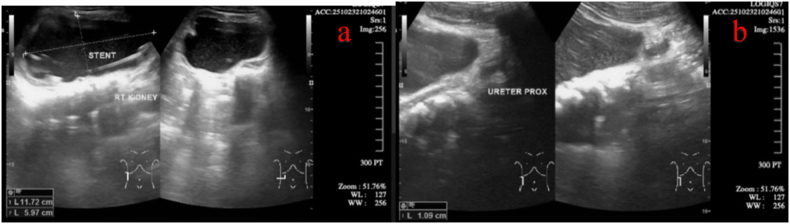
Postoperative abdominal ultrasonography showed that the **(a)** right renal pelvis was still dilated **(b)** without any hydroureter in the right ureter.

Postoperative therapy included intravenous fluids of 0.9 % NaCl at 1000 mL/24 hours, the antibiotic ceftriaxone 600 mg intravenously daily for three days, and analgesics as instructed by the anesthesia team. The Foley catheter was scheduled to be removed on the 10th postoperative day, while the surgical drain was removed if the fluid output was less than 10 mL per day. A ureteral biopsy specimen was sent to the laboratory for histopathology analysis, and the patient was scheduled for a follow-up abdominal ultrasound two weeks postoperatively (October 23, 2025) to assess renal decompression and stent placement, as shown in Fig. 4.

**Fig. 5:**
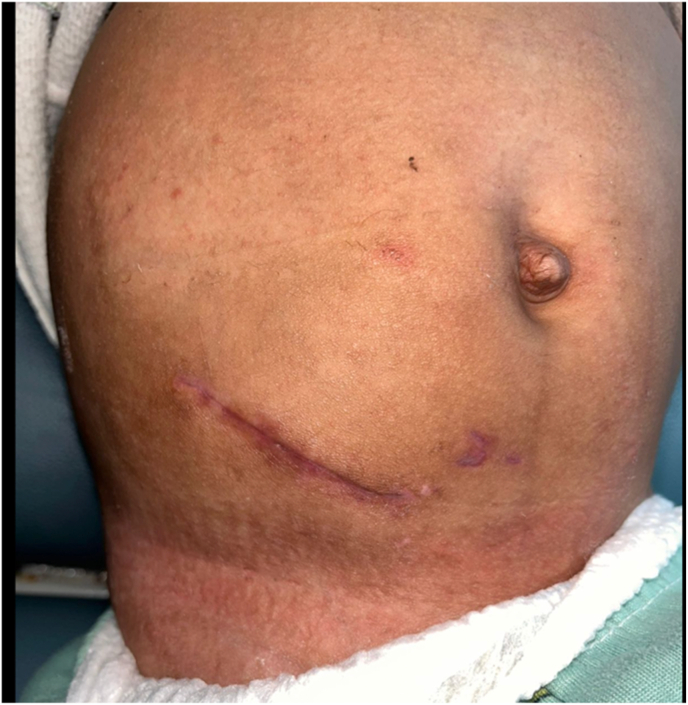
Postoperative wound tissue healing.

The final intraoperative diagnosis was stenosis of the right ureterovesical junction and ureteropelvic junction, accompanied by mid-ureteral stricture, in a case of grade IV hydronephrosis due to multilevel congenital ureteral obstruction, which was successfully treated with ureteral neo-implantation and non-dismembered V–Y type pyeloplasty with DJ stent placement, as shown in Fig. 5.

## 3 Discussion

Congenital ureteral obstruction is a common cause of hydronephrosis in children; however, superimposed obstruction affecting both the ureteropelvic junction (UPJ) and the ureterovesical junction (UVJ) is extremely rare.^4^ Most cases are unilateral and diagnosed antenatally, as in our patient who presented with severe hydronephrosis of the right kidney discovered during a routine prenatal ultrasound. Prompt diagnosis allows for monitoring and staged interventions to preserve renal function and prevent irreversible cortical damage.^5^

The etiology of congenital ureteral obstruction is multifactorial, including abnormal muscle development, impaired peristalsis, or intrinsic fibrosis of the ureteral wall. Histopathological analysis revealed a paucity of circular muscle fibers, increased collagen deposition, and impaired innervation of the affected segments.^1^ In our patient, intraoperative observations revealed segmental narrowing at the level of the UPJ, mid-ureter, and UVJ, indicating diffuse intrinsic ureteral dysplasia, rather than a single localized stricture. This multiple-segment involvement presents a significant reconstructive challenge, as simple excision and end-to-end anastomosis are sometimes insufficient.

Radiological assessment is crucial to delineate the anatomic extent and functional consequences of the obstruction. Integration of ultrasonography, MRI urography, and contrast-enhanced imaging provides comprehensive anatomic characterization and functional evaluation. MRI urography demonstrated severe right-sided hydronephrosis with intra-abdominal organ displacement, suggesting chronic high-grade obstruction.^6^ Imaging also confirmed preserved left renal function, suggesting surgery to preserve the affected kidney.

The primary goal of surgical intervention for congenital ureteral obstruction is to restore normal urine flow, preserve kidney function, and prevent recurrent infections and reflux. The surgical method is determined by the location, length, and characteristics of the obstruction, as well as the degree of preservation of kidney function.

The conventional procedure for ureteropelvic junction obstruction is dismembered pyeloplasty (Anderson–Hynes technique), which involves removal of the stenotic segment and reanastomosis between the renal pelvis and ureter.^7^ In cases with short, diffusely narrowed, or compromised ureters, non-dismembered methods such as V–Y pyeloplasty or Foley Y–V plasty are preferred. This technique allows for lumen dilation without the need for ureteral transection, thus preserving vascular supply. A DJ stent was placed to ensure internal drainage and prevent postoperative stenosis. This reconstruction resulted in adequate urine flow and preserved renal function, as confirmed by postoperative urine output and imaging studies.

Ureteroneocystostomy (ureteral reimplantation) is the treatment of choice for obstruction distal to the ureterovesical junction. This procedure is based on the principle of creating a new, patent bladder entry point and establishing a suitable submucosal tunnel to prevent vesicoureteral reflux, as described by Leadbetter and Politano.^8^ A tunnel length-to-diameter ratio of 5:1 ensures an effective anti-reflux mechanism during bladder contractions. Reimplantation is necessary due to complete distal atresia and a non-functioning native ureteral orifice, necessitating the creation of a new neo-implant.

The theoretical justification for combining V–Y pyeloplasty and ureteroneocystostomy is to treat multisegmental disease within a single ureter. Excision alone can sacrifice significant ureteral length and pose a risk of tension on the anastomosis. Staged reconstruction, after temporary urinary diversion via a nephrostomy, allows decompression and restoration of function before final reconstruction. This approach is supported by modern pediatric urology recommendations that emphasize staged therapy for difficult or extensive ureteral dysplasia.^9^

DJ stents are commonly used to facilitate internal drainage, reduce anastomotic tension, and prevent urinary leakage or restenosis during the healing process. Theoretically, stenting maintains ureteral patency by reducing edema and promoting epithelial regeneration. Postoperative monitoring through imaging and ongoing renal function evaluation are essential to verify successful reconstruction and detect potential obstruction recurrence.^10^

Ureteral neo-implantation is performed to create a new, functional ureterovesical junction, thereby preventing vesicoureteral reflux and distal obstruction.^11^ This technique is particularly important because no patent ureteral orifice was detected during cystoscopy.^3^ DJ stent placement allows internal drainage, accelerates mucosal healing, and prevents leakage or postoperative stricture formation. Postoperatively, the patient demonstrated excellent urine output (4.1 mL/kg/hour), indicating successful drainage and preserved renal function. The absence of fever, wound infection, or leakage indicates a successful recovery. A staged approach—starting with urinary diversion via nephrostomy followed by definitive reconstruction—has proven crucial in enhancing renal recovery prior to complex reconstructive surgery.

This case emphasizes the importance of personalized surgical planning in pediatric urology.^12^ In cases of multilevel ureteral obstruction, a single reconstruction method is rarely sufficient; a combination of pyeloplasty and ureteral reimplantation is often necessary to correct abnormalities in both the proximal and distal segments. This approach aligns with recent evidence showing that early intervention with appropriate reconstruction can lead to better long-term renal function outcomes, particularly in cases of severe hydronephrosis.^13^

## 4 Conclusion

In conclusion, multilevel congenital ureteral obstruction is a rare but surgically correctable cause of hydronephrosis in children. Successful management requires careful imaging-based evaluation, staged interventions, and personalized reconstructive surgery. This case demonstrates that the combination of V–Y pyeloplasty and ureteral neo-implantation is a feasible and effective approach to restoring urine flow and preserving renal function in complex congenital ureteral anomalies.

## CRediT authorship contribution statement

**Gede Wirya Kusuma Duarsa:** Writing – original draft, Resources, Investigation, Funding acquisition, Formal analysis, Data curation, Conceptualization. **Made Bagus Ari Pandita:** Writing – original draft, Project administration, Methodology, Investigation, Formal analysis. **Desak Agung Istri Padma Putri:** Writing – review & editing, Supervision, Methodology, Formal analysis. **Ronald Sugianto:** Writing – review & editing, Validation, Supervision, Project administration.

## Funding sources

This research did not receive any specific grant from funding agencies in the public, commercial, or not-for-profit sectors.

## References

[1] Elsawy M., Fahmy A., Youssef M. (2024). Risk factors influencing the recurrence of obstruction post-pediatric pyeloplasty: a single-center prospective study. Alexandria J Med.

[2] Oktar T., Selvi I., Dönmez M.İ. (2024). What to expect on the long-term follow-up of pediatric pyeloplasty: critical time intervals and risk factors. J Pediatr Surg.

[3] Cobellis G., Bindi E. (2023). Pyeloplasty in children with ureteropelvic junction obstruction and associated kidney anomalies: can a robotic approach make surgery easier?. Children.

[4] Tanash M.A., Bollu B.K., Naidoo R. (2023). Laparoscopic versus open pyeloplasty in paediatric pelvi‐ureteric junction obstruction. J Paediatr Child Health.

[5] Cobangbang M.S., Rivera K.C., Rickard M. (2025). Outcomes of non‐reduction vs reduction pyeloplasty: a systematic review and meta‐analysis. BJU Int.

[6] Nugraheni N.K., Prasetyo R.V., Asmaningsih N. (2013). Management of antenatal hydronephrosis. a report of 2 cases. Folia Medica Indones.

[7] Shao Z., Yang Z., Li J. (2023). Hydronephrosis in pediatric horseshoe kidneys: a comparative analysis of open and laparoscopic pyeloplasty and the influence of obstruction causes. Transl Androl Urol.

[8] Vauth F., Zöhrer P., Girtner F. (2023). Open pyeloplasty in infants under 1 Year—proven or meaningless?. Children.

[9] Ortiz-Seller D., Panach-Navarrete J., Valls-González L. (2024). Comparison between open and minimally invasive pyeloplasty in infants: a systematic review and meta-analysis. J Pediatr Urol.

[10] Sharifiaghdas F., Amini J., Narouie B. (2024). Pediatric pyeloplasty in the poor function kidneys: does surgical success guarantee improvement in renal function? single-center experience and review of literature. Urol J.

[11] Sarhan O., Al Awwad A., Al Otay A. (2021). Comparison between internal double J and external pyeloureteral stents in open pediatric pyeloplasty: a multicenter study. J Pediatr Urol.

[12] Jia J., Meng Q., Zhang M. (2021). A comparative study on the efficacy of retroperitoneoscopic pyeloplasty and open surgery for ureteropelvic junction obstruction in children. Pakistan J Med Sci.

[13] Habibi G.R. (2025). Open pyeloplasty for pediatric ureteropelvic junction obstruction at Herat regional hospital: a retrospective case series. Salamat Acad Res J.

